# Heterogeneity Matters: Different Regions of Glioblastoma Are Characterized by Distinctive Tumor-Supporting Pathways

**DOI:** 10.3390/cancers12102960

**Published:** 2020-10-13

**Authors:** Ivana Manini, Federica Caponnetto, Emiliano Dalla, Tamara Ius, Giuseppe Maria Della Pepa, Enrico Pegolo, Anna Bartolini, Giuseppe La Rocca, Grazia Menna, Carla Di Loreto, Alessandro Olivi, Miran Skrap, Giovanni Sabatino, Daniela Cesselli

**Affiliations:** 1 Institute of Pathology, University Hospital of Udine, 33100 Udine, Italy; enrico.pegolo@asufc.sanita.fvg.it (E.P.); anna.bartolini@asufc.sanita.fvg.it (A.B.); carla.diloreto@uniud.it (C.D.L.); daniela.cesselli@uniud.it (D.C.); 2 Department of Medicine, University of Udine, 33100 Udine, Italy; federica.caponnetto@uniud.it (F.C.); emiliano.dalla@uniud.it (E.D.); 3 Neurosurgery Unit, Department of Neurosciences, University Hospital of Udine, 33100 Udine, Italy; tamara.ius@asufc.sanita.fvg.it (T.I.); skrap@aoud.sanita.fvg.it (M.S.); 4 Institute of Neurosurgery, Fondazione Policlinico Gemelli, Catholic University, 00168 Rome, Italy; giuseppemaria.dellapepa@policlinicogemelli.it (G.M.D.P.); giuseppe.larocca@policlinicogemelli.it (G.L.R.); grazia.menna01@icatt.it (G.M.); alessandro.olivi@policlinicogemelli.it (A.O.); giovanni.sabatino@policlinicogemelli.it (G.S.); 5 Department of Neurosurgery, Mater Olbia Hospital, 07026 Olbia, Italy

**Keywords:** 5-aminolevulinc acid, fluorescence guided surgery, glioma associated stem cells, glioblastoma microenvironment, tumor supporting signature

## Abstract

**Simple Summary:**

5-ALA Fluorescence Guided Surgery aims at extending the boundaries of glioblastoma (GBM) resection. It is based on the use of a fluorescent dye, 5-aminolevulinic acid (5-ALA). Depending on the fluorescence levels, it is possible to distinguish the core of the tumor, the infiltrating borders and the healthy tissue. Since GBM progression is supported by tumor cells and their interaction with the surrounding microenvironment, we hypothesized that 5-ALA intensity could identify microenvironments with different tumor supporting properties. Taking advantage of glioma-associated stem cells; a human in vitro model of the glioma microenvironment, we demonstrate that all regions of the tumor support the tumor growth, but through different pathways. This study highlights the importance of understanding the TME to obtain key information on GBM biology and develop new therapeutic approaches.

**Abstract:**

The glioblastoma microenvironment plays a substantial role in glioma biology. However, few studies have investigated its spatial heterogeneity. Exploiting 5-ALA Fluorescence Guided Surgery (FGS), we were able to distinguish between the tumor core (ALA+), infiltrating area (ALA-PALE) and healthy tissue (ALA−) of the glioblastoma, based on the level of accumulated fluorescence. The aim of this study was to investigate the properties of the microenvironments associated with these regions. For this purpose, we isolated glioma-associated stem cells (GASC), resident in the glioma microenvironment, from ALA+, ALA-PALE and ALA− samples and compared them in terms of growth kinetic, phenotype and for the expression of 84 genes associated with cancer inflammation and immunity. Differentially expressed genes were correlated with transcriptomic datasets from TCGA/GTEX. Our results show that GASC derived from the three distinct regions, despite a similar phenotype, were characterized by different transcriptomic profiles. Moreover, we identified a GASC-based genetic signature predictive of overall survival and disease-free survival. This signature, highly expressed in ALA+ GASC, was also well represented in ALA PALE GASC. 5-ALA FGS allowed to underline the heterogeneity of the glioma microenvironments. Deepening knowledge of these differences can contribute to develop new adjuvant therapies targeting the crosstalk between tumor and its supporting microenvironment.

## 1. Introduction

Glioblastoma multiforme (GBM) is the most common and aggressive primary tumor affecting the adult central nervous system, characterized by a rapid and unavoidable lethal prognosis [1,2]. In fact, despite current therapies based on maximal surgical resection combined with concomitant radio and chemotherapy, GBM patients show a median survival time of less than 2 years [3].

The failure of therapies is partly due to the impossibility to achieve a radical resection, as cancer cells tend to escape the tumor site and infiltrate the adjacent brain, thus causing recurrences a few months after surgery [4]. Accordingly, many studies have shown that, in newly diagnosed GBM, the strongest prognostic factor, for both overall survival (OS) and disease free survival (DFS), is the extent of resection (EOR) [5,6,7,8,9].

However, discriminating normal brain from tissue infiltrated by tumor cells is difficult, even for experienced neurosurgeons, adopting surgical microscopy. To overcome this limitation and maximize the EOR, in the past decades, advanced techniques, such as neuro-navigation and intraoperative imaging, were initiated to support the intraoperative identification of tumor and tumor borders [10,11]. More recently, Fluorescence Guided Surgery (FGS) was introduced as a promising method in the management of GBM [12]. FGS relies on the use of fluorescent dyes, such as 5-ALA (5-aminolevulinic acid), that selectively accumulate within tumor cells and become visible to the surgeon, giving a real-time information on tumor presence directly from the tissue and not from conveyed images [13,14]. 5-ALA, an endogenous intermediate in the synthesis of heme group, is accumulated, as protoporphyrin IX (PpIX), specifically in tumor cells as a result of the dysregulation of the heme’s biosynthetic pathway [15]. Since its approval, many studies have reported the benefits following the introduction of 5-ALA FGS, alone or in conjunction with other surgical techniques, in terms of both increased extent of resection and patient survival [16,17,18,19].

As the accumulation of the dye is the direct reflection of the tumor cell concentration, it allows one to distinguish areas with different fluorescence levels, going from bright to pale to negative. Histopathological studies have shown that these areas (bright, pale and negative) correspond to tumor mass, the infiltrated margins and the healthy brain, respectively [20]. Transcriptomic analyses showed an increased expression of ABCG2, ALDH1, FGF1 stem cell markers in the pale region, when compared to the tumor mass, suggesting the presence, in the infiltrating front of the tumor, of a glioma stem cell (GSC) population possibly responsible for tumor recurrence [21].

However, GBM growth, progression and drug resistance are not only supported by GSC and tumor cells but also by their interaction with the surrounding environment, defined as tumor microenvironment (TME) [22,23,24,25]. The latter contains various types of non-tumor cells, such as endothelial cells, stromal cells, pericytes, immune cells, and component of the extracellular matrix (ECM), primed by tumor cells to promote proliferation, invasion, angiogenesis and immune suppression, well known hallmarks of cancer [26,27,28,29]. In this process a crucial role is played by the inflammation sustained by innate immune cells. These latter, through the release of bioactive molecules, including growth factors, survival factors, proangiogenic factors and enzymes, lead to the activation of the TME thus initiating other hallmark-promoting programs [30].

In this regard, we have previously established a protocol to isolate from human gliomas a population of stem cells, named glioma-associated stem-cells (GASC), representing a patient-based model of the glioma microenvironment. Indeed, GASC are devoid of tumor-initiating properties, but show stem cell properties and the ability to support the, in vitro, biological aggressiveness of tumor cells [30,31,32]. We also reported that the transcriptomic profile of GASC is prognostic in low grade gliomas [33].

Therefore, to investigate whether different regions of gliomas are characterized by distinctive tumor supporting properties, we utilized GASC isolated from regions showing strong (ALA+), pale (ALA PALE) or no fluorescence for 5-ALA (ALA−) of patients, undergoing to 5-ALA fluorescent guided surgery for a newly diagnosed GBM.

Here we report that GASC derived from ALA+, ALA PALE and ALA− regions, while sharing a similar phenotype, were characterized by a different gene expression profile. Interestingly, these differences were also detected at tissue level interrogating the TCGA database. Finally, a “five gene immune-related signature”, defined studying the ALA+ GASC, resulted to be predictive of OS and DFS. Deepening the knowledge of the spatial heterogeneity of the pathways supporting glioma growth and immune escape could help in improving adjuvant strategies aimed at targeting the crosstalk between tumor cells and TME.

## 2. Results

### 2.1. Glioma-Associated Stem Cells Were Efficiently Isolated from the Regions with Different 5-ALA Positivity

Samples representative of regions with strong (ALA+), pale (ALA-PALE), or no 5-ALA-fluorescence (ALA−) were obtained from six patients undergoing 5-ALA FGS for a newly diagnosed GBM. 

**Figure 1:**
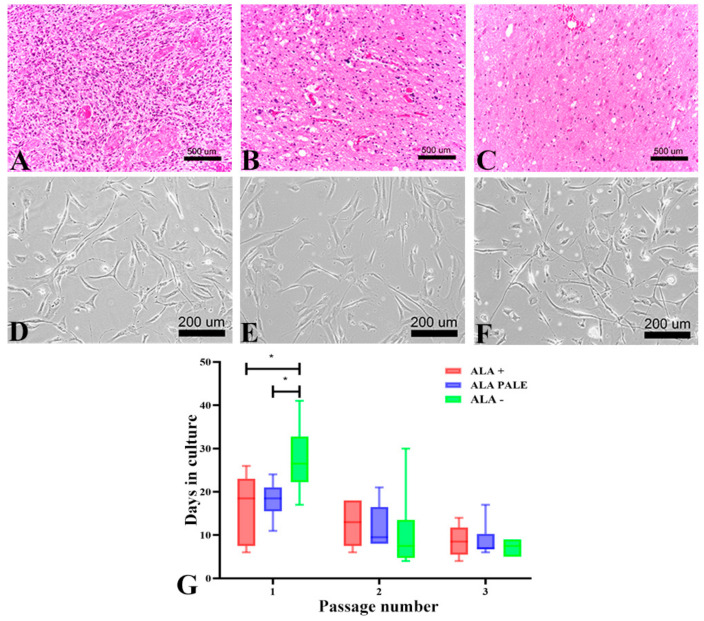
GASC isolated from tumor regions showing different intraoperative 5-ALA fluorescence intensity display different growth kinetics. (**A**–**C**) Pictures of HE-stained sections representative of 5-ALA+, 5-ALA PALE and 5-ALA− regions of a newly diagnosed GBM removed by 5-ALA FGS. (**A**) The 5-ALA+ region displays features of a high-grade tumor (high cell density and mitotic activity, nuclear pleomorphism and necrosis). (**B**) The 5-ALA PALE section is characterized by a gliotic tissue infiltrated by tumor cells. (**C**) The 5-ALA− section consists of brain tissue devoid of cancer cells (**D**–**F**) Contrast phase images of GASC isolated from 5-ALA+ (**D**), 5-ALA PALE (**E**) and 5-ALA− (**F**) regions showing, in vitro, a similar fibroblast-like morphology, typical of adult mesenchymal stem cells. (**G**) The histograms represent the growth kinetic of GASC-ALA+, GASC-ALA PALE and GASC-ALA−, as evaluated at the first, second and third passage in culture. Values were calculated by counting days between passages. Data are presented as interleaved box and whiskers (*n* = 6). * *p* value < 0.05. Statistical analysis was performed by a Student’s *t*-test (GraphPad Prism 8).

ALA+ tumor specimens were almost always characterized by a diffusely infiltrative high-grade glial tumor with very high cell density, remarkable nuclear pleomorphism, high mitotic activity; furthermore, areas of necrosis (even focally) and microvascular proliferation were present (Figure 1A). 

Samples with pale fluorescence were characterized by gliotic tissue, focally infiltrated by a low-to-moderate cell dense glial tumor, with low-to-high nuclear pleomorphism, none-to-few mitotic figures, absence of necrosis and microvascular proliferation (Figure 1B). No distinct tumor cells were identified in the majority of samples obtained from ALA− regions (Figure 1C).

Despite their different histological aspects, GASC were efficiently isolated from each of the three regions (Figure 1D–F). However, GASC isolated from ALA− regions took significantly longer time to reach confluence, compared to those isolated from the respective ALA+ and ALA-PALE regions (mean time of 27 days vs. 16 days vs. 18 days, respectively; Figure 1G). As the number of passages increased, the cultured cells acquired a similar homogeneous fibroblast-like morphology (Figure 1D–F) and reached the same growth kinetic (Figure 1G). No major differences were seen among GASC isolated from the six GBM tissues.

### 2.2. GASC Isolated from the Three Different 5-ALA Positive Regions Share a Similar Phenotype

**Figure 2:**
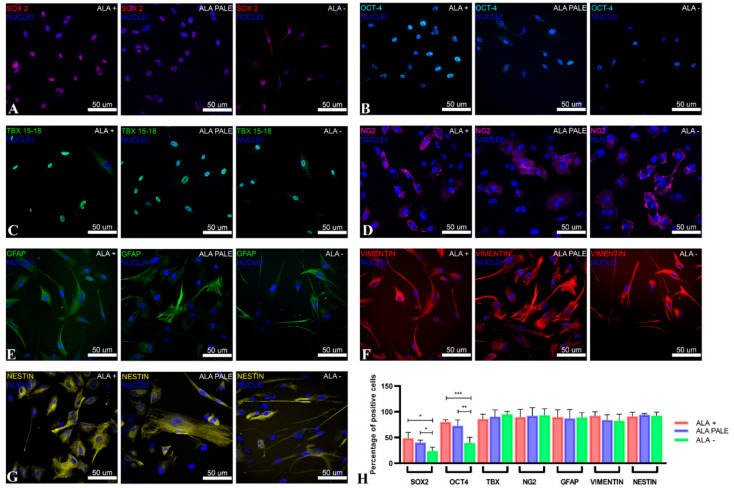
Phenotypic characterization of GASC isolated from 5-ALA+, 5-ALA PALE and 5-ALA− regions. (**A**–**C**) The expression of the pluripotent state-specific transcription factors Sox2 (red fluorescence, (**A**)) and Oct4 (cyan fluorescence, (**B**)) progressively decreases in GASC obtained from ALA+ (left image), ALA PALE (middle image) and ALA− regions (right image). No differences were detected in TBX expression (green fluorescence, (**C**)). (**D**–**G**) Cytoplasmic protein expression. Expression of, NG2 (magenta fluorescence, (**D**)), GFAP (green fluorescence, (**E**)), vimentin (red fluorescence, (**F**)) and nestin (yellow fluorescence, (**G**)) did not differ among GASC obtained from different tumor regions. Nuclei are depicted by the blue fluorescence of DAPI staining. Images were acquired using the confocal microscope Leica TCS-SP8 (Leica, Wetzlar, Germany). (**H**) Histograms represent the fraction of cells expressing the assayed markers. Data are presented as mean ± standard deviation (*n* = 6). Statistical analysis was performed by repeated measurements one-way ANOVA followed by Tukey post-test, * *p* value < 0.05; ** *p* value < 0.001; *** *p* value < 0.0001.

At the third passage in culture, GASC cells were evaluated for the expression of intermediate filaments as well as stem cell- and pericyte-derived markers. As shown in Figure 2, cells isolated from the different 5-ALA positive regions did not differ in the expression of GFAP (Figure 2E), vimentin (Figure 2F) and nestin (Figure 2G), nor in the expression of markers such as TBX and NG2 (Figure 2C,D, respectively), which are known to characterize neural precursors and pericytes, respectively. Regarding the stem cell transcription factors SOX2 (Figure 2A) and OCT4 (Figure 2B), GASC isolated from the ALA+, as well as ALA-PALE, regions showed a significantly higher expression of these markers compared to those isolated from the corresponding ALA samples (Figure 2H). This confirms that 5-ALA is able to efficiently discriminate a region rich in stem cells and indicates that the ALA-PALE region, an extremely interesting area because it represents the rims of the tumor, hosts a population with a stem-like phenotype.

**Figure 3:**
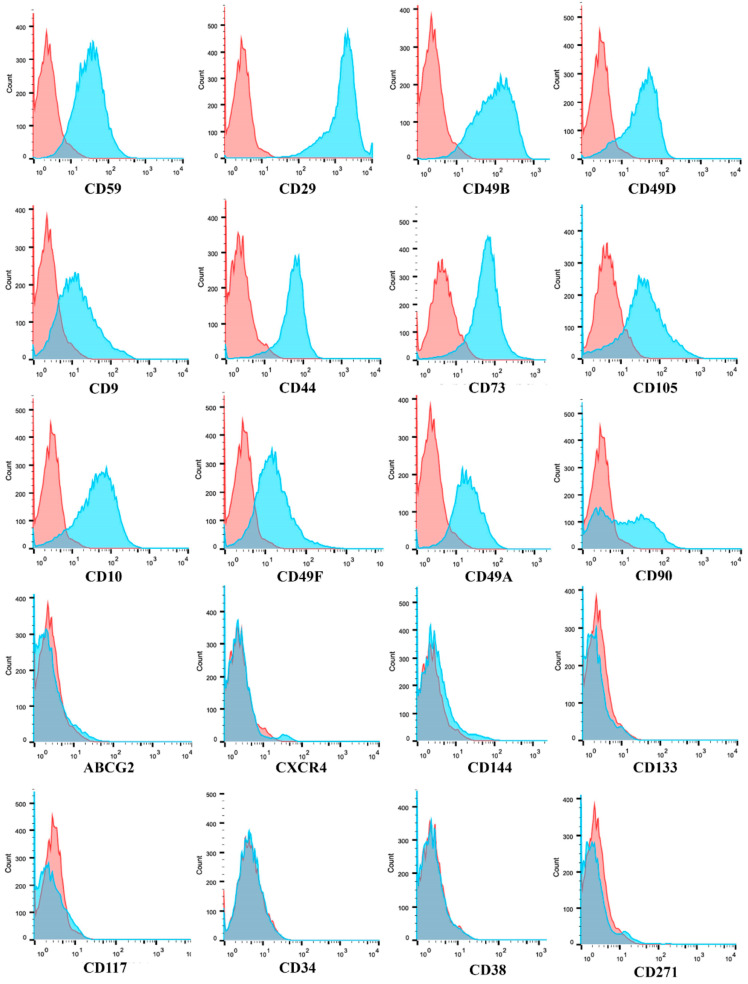
GASC-ALA+, ALA PALE, ALA− share the same mesenchymal stem cell immunophenotype. Representative histograms of the flow cytometry analysis of GASC cell populations. Histogram overlays show isotype control IgG staining profile (red histogram) versus the specific antibody staining profile (cyan histogram). The expression of twenty surface antigens was evaluated using the flow cytometer FACS CANTO II (BD Biosciences). See Table 1 for quantitative data.

Flow cytometry analysis, as shown in Table 1 and Figure 3, displayed that GASC isolated from the different 5-ALA positive regions shared the same mesenchymal immunophenotype. Specifically, GASC highly expressed integrins such as CD29, CD49b, CD49d, CD49a and CD49f, and canonical mesenchymal stem cell markers, such as CD44, CD73, CD105, CD44 and CD90. Moreover, consistently with a mesenchymal stem cell phenotype, GASC reported low/negative levels of CD144, ABCG2, CXCR4, CD117, CD34 and CD38. Low expression of CD271 was also observed (Table 1).

Our data suggest that GASC, regardless their distance from the tumor core, share similar phenotypic properties. Importantly, independently from the region of origin, no major differences were seen among GASC isolated from the six GBM patients.

### 2.3. GASC Isolated from the Three Different 5-ALA Positive Regions Present Distinct Gene Expression Signatures

**Figure 4:**
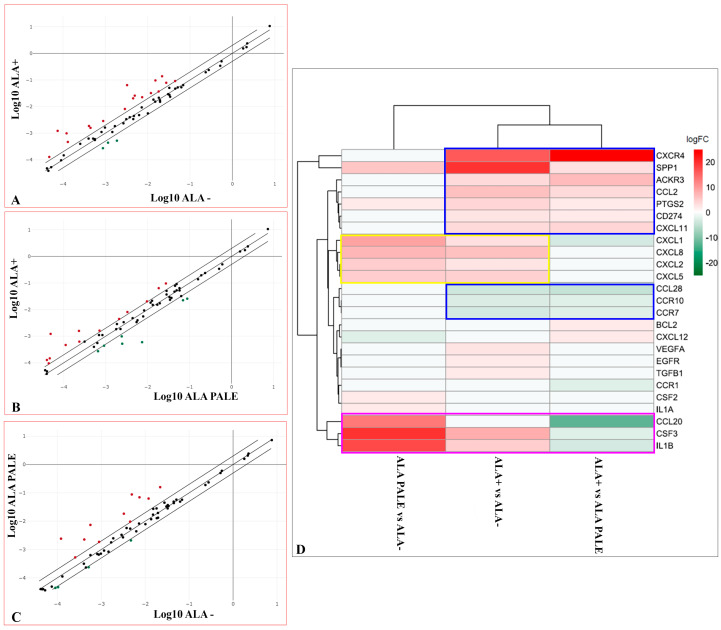
Comparison of the gene expression profile of GASC-ALA+, ALA PALE and ALA− using the cancer inflammation and immunity crosstalk RT^2^ PCR Array. (**A**–**C**) Scatter plots comparing the expression of eighty-four cancer-related genes analysed using the RT2 Profiler™ PCR Array. Scatter plots represent the mean of normalized expression of every gene between ALA+ vs. ALA− (**A**), ALA + vs. ALA-PALE (**B**) and ALA-PALE vs. ALA− (**C**), respectively (*n* = 6 per region). Using as a threshold a fold change ≤−2 or ≥2 (dotted lines), upregulated (red dots) and down-regulated (green) genes resulted to be, respectively, 18 and 3 in ALA+ vs. ALA− comparison (**A**), 13 and 7 in ALA+ vs. ALA− (**B**) PALE comparison and 11 and in 4 ALA-PALE vs. ALA− (**C**) comparison. (**D**) Heatmap of the RT^2^ Profiler gene expression results showing the 25 differentially expressed genes in the ALA+/ALA PALE/ALA− comparisons, represented as the mean value of Log2Fold Change calculated for each gene, in 6 different GASC cell lines for each region. Hierarchical clustering of genes and samples was performed using Euclidean distance and the complete agglomeration method. The heatmap was generated using the heatmap R/Bioconductor package.

Although the phenotypic characterization of GASC isolated from the three different 5-ALA positive regions did not reveal considerable differences, we hypothesized that diverse proximity of the TME to the tumor bulk could differently imprint on the genetic profile of the microenvironment. For these reasons, the expression of 84 key genes involved in modulating the communication between tumor cells and the cellular mediators of inflammation and immunity in the TME was evaluated using the Human Cancer Inflammation and Immunity Crosstalk RT^2^ Profiler PCR Array. In fact, it is well known that glioma cells, through the secretion of molecules, such as pro-inflammatory cytokines and chemokines, could be able to establish an immunosuppressive microenvironment and reprogram non-tumor elements to became supportive for tumor progression [34,35,36,37]. Figure 4A–C show scatter plots that compare, two by two, GASC of the three regions two by two, in which are highlighted the genes with a fold change greater than 2 (red dots) or less than −2 (green dots). 

For most of these genes, listed in Table 2, Table 3 and Table 4, the difference in expression was indeed statistically significant. 

Considering the 25 genes differentially regulated in at least one of the three comparisons and applying an unsupervised hierarchical clustering, it was apparent a greater analogy in the genes differently expressed between ALA+ vs. ALA− and ALA+ vs. ALA PALE (Figure 4D). In fact, CXCR4, SPP1, ACKR3, CCL2, PTGS2, CD274 (PD-L1), CXCL11 were upregulated while CCL28, CCR1, CCR7 were down regulated in both comparisons (Figure 4D, blue boxes), suggesting this as a signature specific of the core region of the tumor. 

Two other interesting groups of genes were identified. ALA-PALE and ALA+ derived GASC highly expressed, with respect to those isolated from ALA− region, the C-X-C motif chemokines CXCL1, CXCL2, CXCL5 and CXCL8 (IL8) (Figure 4D, yellow box), implying the presence of some shared features between ALA+ and ALA-PALE GASC. Moreover, the ALA-PALE region was characterized by an overexpression, with respect to both ALA+ and ALA−derived GASC, of CCL20, CSF3 and IL1B (Figure 4D, magenta box), suggesting the possible importance of these three genes in the infiltrating front of the tumor.

As a whole, the transcriptomic analysis showed that ALA-PALE and ALA+ derived GASC, although sharing the expression of some genes (Figure 4D, yellow box), were characterized by different gene signatures.

### 2.4. An Immune-Related Gene Signature, Based on GASC Study, Could Predict GBM Prognosis

**Figure 5:**
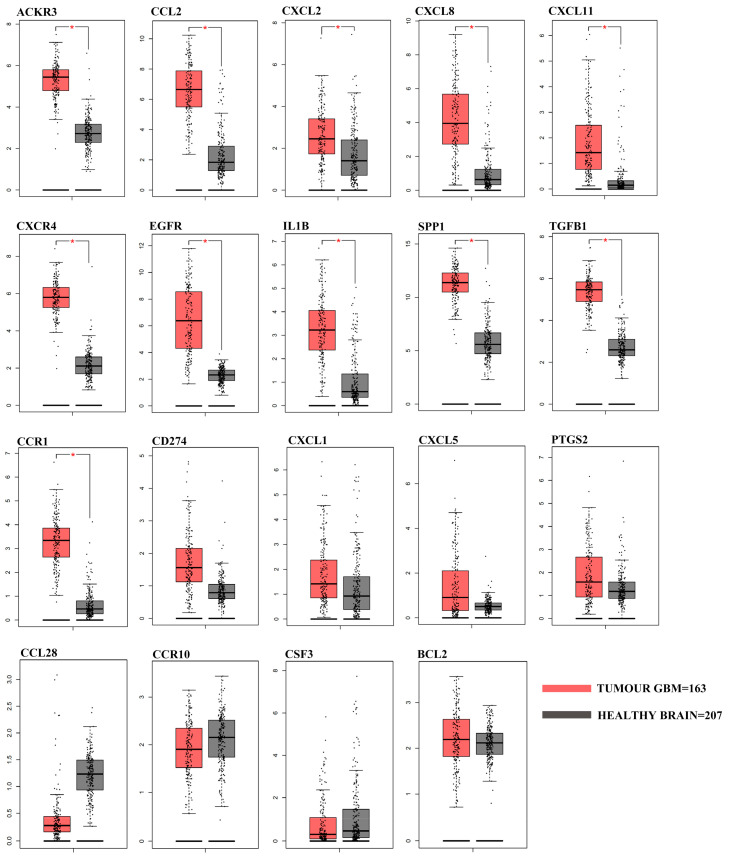
Differential gene expression analysis in the TCGA-GBM RNA-seq dataset. Box plots represent the gene expression levels in the TCGA-GBM dataset (*n* = 163) and in the TCGA-GTEx matched normal samples (*n* = 207) of the 19 differentially expressed genes identified with the RT2 Profiler array. Data was obtained and plotted from the GEPIA web server (|Log2FC| Cutoff: 1; * *p* value < 0.01).

In order to understand if the differentially expressed genes identified by studying GASC could have a clinical relevance, we evaluated their expression in 163 GBM included in the Cancer Genome Atlas GBM dataset (TCGA-GBM). In particular, we queried the GEPIA web server comparing the TCGA RNA-Seq data of GBM patients with that of 207 TCGA/GTEx matched normal samples. We confirmed that eleven out of fifteen genes differentially expressed in ALA+ versus ALA− GASC (ACKR3, CCL2, CXCL11, CXCL2, CXCL8, CXCR4, EGFR, IL1B, SPP1,TGFB1 and CCR1) were also upregulated in TCGA tumor patients, while the remaining four (CXCL1, CXCL5, PD-L1 and PTGS2) showed a similar, but not statistically significant, trend (Figure 5).

**Figure 6:**
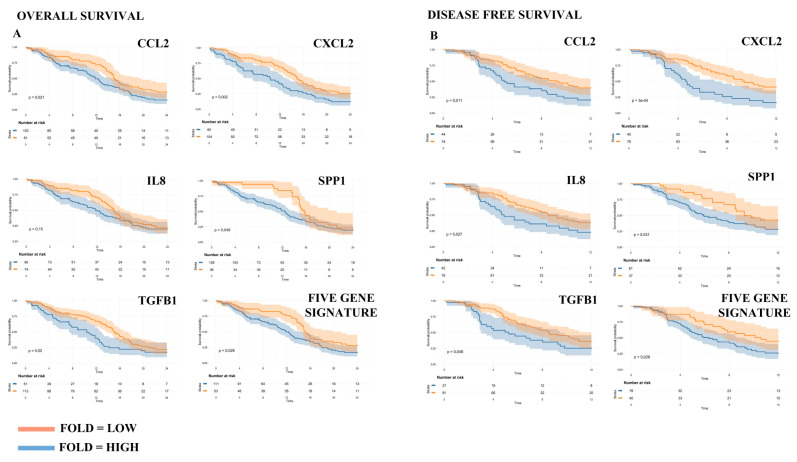
An immune-related gene signature, based on GASC study, could predict GBM prognosis. The prognostic value of CCL2, CXCL2, IL8, SPP1 and TGFB1 was assessed independently in the TCGA-GBM RNA-seq dataset and was represented by Kaplan-Meier plots. Both the (**A**) Overall Survival (OS, left panel) and the (**B**) Disease-Free Survival (DFS, right panel) were evaluated. Afterwards, the analysis was repeated considering the five-genes signature (median expression). Patients were stratified based on the optimal cut-point and data were censored at 24 months for OS and at 12 months for DFS.

Afterwards, we applied the Cox Proportional-Hazards Model [38] to estimate which genes were associated with Disease-Free Survival (DFS, defined as the time between cancer diagnosis and local or distant relapse) and Overall Survival (OS, defined as the time between cancer diagnosis and death for any cause), censoring data at 12 and 24 months, respectively (Figure 6). Interestingly, five genes (CCL2, CXCL2, CXCL8, SPP1 and TGFB1), when highly expressed, were significantly associated with reduced DFS and OS (Table 5). Moreover, these five genes taken together retained their prognostic value for both DFS and OS (Table 5 and Figure 6) and, actually, this gene signature often outperformed single genes, especially with respect to OS (Table 5). Altogether these results support the clinical value of GASC and their possible usefulness to infer information on the role of the microenvironment in the different GBM regions.

## 3. Discussion

The infiltrative nature of tumor cells and their wide cellular heterogeneity are considered among the major causes of treatment failure in GBM [39,40]. Both features are partly derived from the complex interactions that GBM cells establish with the surrounding TME, comprising stromal cells, immune cells, vessels, and extracellular matrix components. This crosstalk, mediated by different routes of information exchange, such as cell-cell contact, release of soluble factors or bioactive molecules enclosed in extracellular vesicles, support GBM growth, infiltrative spread and immune escape [4]. Therefore, studying the human GBM microenvironment could help understand GBM biology and possibly the development of novel therapeutic approaches or why the standard ones fail [41]. However, while many studies have investigated the heterogeneity of tumor cells, especially glioma stem cells, very few have focused on the tumor microenvironment. This because of the difficulty in obtaining human model of the TME representative of the different GBM regions.

5-Aminolevulinic acid (5-ALA), the most common fluorescent molecule used in Fluorescence Guided Surgery (FGS), was approved in Europe and in the United States in 2007 and 2017, respectively [13,14]. Its specific accumulation, as protoporphyrin IX (PpIX), in tumor cells, allows detecting not only the tumor mass, but also the infiltrating areas, not detectable using contrast-enhanced MRI, thus representing a potent tool to extend the boundaries of the GBM resection. Indeed, its efficacy in improving patient outcome has been shown [16,17,18,19]. We wanted to take advantage of this model to identify and sample the core region of the tumor, the infiltrating front and the healthy tissue, characterized, respectively, by a bright fluorescence (ALA+), by a dim fluorescence (ALA-PALE) and by no fluorescence (ALA−).

So far, ALA+ and ALA-PALE regions have been studied in terms of histological features or by isolating and characterizing GSC [42,43]. More recently, a proteomic study identified 37 proteins differentially expressed across the different 5-ALA positive tissues, observing a higher expression of anti-apoptotic and pro-survival proteins in the tumor margins, whereas proteins regulating DNA damage responses were overexpressed in the tumor core [44]. However, a comprehensive characterization of microenvironmental cells residing in different tumor areas is still lacking.

In light of this, and taking advantage of 5-ALA FGS, we exploited a human in vitro model of the glioma microenvironment, represented by glioma-associated stem cells (GASC), to identify whether, moving from the core of the tumor towards its infiltrating front, the phenotype and gene expression profile of GASC change.

As mentioned, GASC were previously isolated from human low- and high-grade gliomas and represent, bona fide, a population of glioma stromal cells, residing in the tumor microenvironment, characterized by stemness features and tumor-supporting function [30,31,32]. We also reported that, in low grade gliomas, GASC can predict prognosis thus representing a reliable patient-based model [33].

In the present study we showed that GASC were isolated from ALA+, ALA PALE and ALA− regions with the same efficiency, but GASC derived from ALA− regions had a longer lag time in culture, suggesting a reduced activation state. Moreover, GASC isolated from the three different 5-ALA positive regions exhibited a similar mesenchymal undifferentiated phenotype, as assessed by immunofluorescence and flow-cytometry. However, comparing the ALA+ and the ALA PALE regions with the ALA− we found a higher percentage of Sox2 and Oct4 positive cells, indicating that cells endowed with stemness and able to support the glioma growth were located also beyond the tumor mass, in the region defined by a vague 5-ALA fluorescence, that cannot be eliminated, during surgery, without the support of the fluorescent stain. Previous studies, based on transcriptomic analysis, showed an increased expression of ABCG2, ALDH1 and FGF1 stem cell markers in the pale region, when compared to the tumor mass [21], indicating the presence, in the infiltrating front of the tumor, of a glioma stem cell (GSC) population possibly responsible for tumor recurrence [22,23,24]. Our data suggest that an increase in stemness properties could also be present in the microenvironment.

Glioma cells are able to corrupt the TME to trigger inflammatory responses or to establish an immunosuppressive environment. Using a pre-defined commercially available panel, we profiled the expression of 84 genes related to inflammation and cancer immunity, in GASC isolated from the 5-ALA+, ALA pale and ALA− regions. Comparing GASC of the different regions (ALA+ vs. ALA−, ALA+ vs. ALA-PALE, ALA-PALE vs. ALA−) we confirmed that they were characterized by a different gene signature and we could recognize four major groups of genes:

(1) Genes preferentially upregulated in GASC obtained from ALA+;
(2) Genes upregulated in GASC derived from either ALA+ or ALA-PALE regions;
(3) Genes preferentially upregulated in GASC derived from ALA-PALE fragments;
(4) Genes preferentially upregulated in GASC obtained from ALA-PALE and ALA− regions.

The first group comprised CXCR4, SPP1, ACKR3, CCL2, PTGS2, CD274 [PD-L1), CXCL11 and suggests that the core region of GBM is characterized by signaling pathways strictly related to hypoxia and strongly favoring stemness and immune tolerance through PD-L1 overexpression. Indeed, CXCR4 is a G-protein coupled receptor with only two known ligands, the chemokines SDF-1 (also known as CXCL12) and macrophage migration inhibitory factor (MIF). SDF-1 and CXCR4 play a role not only in the epithelial-to-mesenchymal transition (EMT) and in the generation and self-renewal of cancer stem cells, but also in generating and maintaining the perivascular stem cell niche, regulating the migration of GSC along the pre-existing vasculature and in inducing chemo- and radio-resistance [45,46]. Atypical Chemokine Receptor 3 (ACKR3), as CXCR4, is a G protein-coupled receptor belonging to the CXC chemokine receptor family [47]. It can bind both CXCL12 and CXCL11 and heterodimerize with CXCR4 and its effect can vary. Indeed, ACKR3 can inhibit tumor growth and progression by acting on chemokine bioavailability, or it can promote tumorigenesis by regulating the CXCR4 signaling [48]. Its role in glioma is now emerging [49]. ACKR3 expression is increased in GBM, especially in tumor cells and vessels, and correlate with a bad prognosis. Additionally, it has been shown, in murine models of GBM, that the combined use of temozolomide with a chimeric antibody against ACKR3/CXCR7 could activate an immune response and improve survival [50]. Secreted Phosphoprotein 1 (SPP1, also known as osteopontin), is involved in tumorigenesis, acting particularly in remodeling the extracellular matrix, thus modulating proliferation, invasion, metastasis and angiogenesis, and its expression has been correlated with poor prognosis in different tumors [51,52,53]. In GBM, SPP1 is secreted by healthy stromal astrocytes and in the perivascular niche of proneural GBM, where it promotes stemness and radio resistance in tumor cells [54]. In vitro and in vivo cancer models showed that the induction of a stem like phenotype by SPP1 results in aggressive recurrent gliomas, by acting on Wnt signaling, cell cycle and focal adhesions [55]. High expression of SPP1 is also found in invasive mesenchymal GBM and correlated with high levels of vimentin and low levels of GFAP. Accordingly, down-regulation of SPP1 reduced tumor cells invasion and vimentin expression, but increased the level of GFAP, a marker of differentiated astrocytes [56]. C-C Motif Chemokine Ligand 2 (CCL2, also known as monocyte chemotactic protein 1), was demonstrated to be involved in the progression of several cancers by activating PI3K/Akt, Rac GTPase and p42/44 MAPK signaling pathway [57,58] and by recruiting different subsets of myeloid cells [59,60]. In the glioma microenvironment, CCL2, released by macrophages and microglia, is crucial for attracting myeloid-derived suppressor cells (MDSCs) and Tregs thus creating an immunosuppressive/tumor supporting environment [61]. Similarly, prostaglandin-endoperoxide synthase 2 (PTGS2, also known as cyclooxygenase-2), is mainly expressed by glioma cells, macrophages and microglia surrounding areas of tumor necrosis and, through the release of prostaglandin E2, induces GBM cell proliferation and invasion, angiogenesis, immune suppression, and immune evasion [62]. Finally, CD274, also known as programmed death-ligand 1 (PD-L1), resulted up-regulated in ALA+-derived GASC. The axis PD1/PDL1 is the target of many checkpoint inhibitors aimed at reverting the immune-tolerant signaling that occurs in tumors. In GBM, PD-L1 expression, both at protein [63] or mRNA level [64], acts as a negative prognostic factor. The upregulation of PD-L1 in GBM cells and microglia is induced by the activation of various receptors such as toll like receptor (TLR), epidermal growth factor receptor (EGFR), interferon alpha receptor (IFNAR) and interferon-gamma receptor (IFNGR) [65]. The overexpression of PD-L1 promotes immunosuppression by inducing the expansion of T regulatory cells [66] able to inhibit the proliferation of CD4+ and CD8+ effector T-cells, as elegantly demonstrated by Di Domenico’s group [67].

The second group of genes, overexpressed in GASC from either ALA+ or ALA-PALE regions, included C-X-C motif chemokine ligand (CXCL1), CXCL2, CXCL5 and CXCL8, all ligands of CXCR2. In this case, the identification of a shared group of genes involved in tumor invasion and angiogenesis in both ALA+ and ALA PALE regions underlines the presence, in the pale region, of a signature promoting tumor invasion and angiogenesis. In fact, CXCL8, commonly known as IL8, and CCL2 are actively expressed in the mesenchymal subtype of GBM, where they play different and cooperative roles in promoting proliferation, invasion, angiogenesis and macrophage polarization, under the control of NFKB-dependent STAT3 activation pathway [68]. Accordingly, in PTEN-deficient GBM, the inhibition of STAT3 allows the expression of CXCL8 gene that promotes proliferation, invasion and spreading of glioma cells [69]. Indeed, although in most tumors CXCL8-CXCR1/CXCR2 signaling is correlated with induction of angiogenesis [70], in GBM CXCL8 correlates with cell proliferation and invasiveness. Additionally, CXCL8 was described as a key player in the drug-induced acquisition of chemoresistance: treatment with temozolomide induces an epigenetic change in the promoter of CXCL8 with its consequent overexpression. This phenomenon alters the phenotype of GBM cells, shifting them to a less differentiated GSC-like state, responsible for recurrence and chemoresistance [71]. CXCL2 is another ligand of CXCR2 and, in recent reports, the axes CXCL2-CXCL8/CXCR2 is described as an important signaling pathway modulating GBM angiogenesis, alternative to the well-known VEGF-VEGFR one, thus representing a new target for glioma therapy. In particular, in an in vivo mouse model of GBM, during the early phase of tumor initiation, it was observed the accumulation and proliferation of resident microglia IBA-1+ cells in the perivascular niche. These cells, through the release of CXCL2 in the microenvironment, participate in the building and stabilization of new tumor vessels. In fact, depleting the tumor of microglia or blocking CXCR2 on endothelial cells, significantly reduced glioma volume [72,73]. Likewise, CXCL1 and CXCL5 are interactors of CXCR2. The role of CXCL1 in glioma is poorly described, however in breast and colorectal cancer it is depicted as promoter of metastasis [74] and involved in the formation of the premetastatic niche [75]. Moreover, in ovarian cancer, stromal cells, surrounding the tumor, release CXCL1, CXCL2 and CXCL8 exerting a chemoprotective role on cancer cells and contributing to polarize monocyte/macrophage toward an M2 tumor promoting phenotype [76]. Emerging role of CXCL5 is found in glioma biology since its expression has been linked with poor patient prognosis and, in vitro, CXCL5 increased migration and proliferation of the glioblastoma cell line U87 [77].

The third group of genes, selectively up-regulated in the ALA-PALE-derived GASC, included CCL20, CSF3 and IL1b. This signature, related to activated astrocytes and microglial cells, enhances the HIF-1-mediated hypoxic response and favors immunosuppression, by inducing lymphopenia and diverging monocyte phenotype towards a myeloid-suppressor phenotype.

In fact, in a glioma model, it has been shown that CCL20 [chemokine C-C motif ligand 20) is released by astrocytes in response to hypoxia and, by stimulating the CCR6-NF-κB signaling pathway, further enhances the HIF-1-mediated hypoxic response [78]. Additionally, in humans, GBM expression of CCL20 and its receptor CCR6 predicts poor prognosis and plays a crucial role in immunomodulation [79]. Regarding colony stimulating factor 3 (CFS3, also known as granulocyte colony stimulating factor or G-CSF), it has been shown that, in the presence of tumor, brain cells produce CSF3 and this production shift hematopoiesis toward granulocytic lineages, causing lymphopenia and favoring immunosuppression [80]. Additionally, CSF3 promoted, in vitro, proliferation, migration and invasion of glioma cells [81,82]. Finally, IL1b can be released by brain microglia and infiltrating peripheral macrophages and promotes tumor growth, endothelial cell activation, tumor angiogenesis and further induction of immunosuppressive cells, through NF-kB and MAPK pathways [83].

Finally, the fourth group of genes included CCL28 and its receptor CCR10, both up-regulated in ALA PALE and ALA− GASC, with respect to the ALA+. In ovarian cancer, the overexpression of CCL28 promotes the recruitment, to the tumor site, of the Treg cells, through their CCR10 receptor. Here, Treg cells promote tumor tolerance and are a source of VEGF, thus contributing to angiogenesis [84]. Similarly, the interaction between CCL28/CCR10 is described as responsible for the remodeling of lymphatic vessels, increasing the metastatic potential of cancer cells [85]. Here we describe that CCL28/CCR10 axis could be active outside the tumor core and that it could represent a connection between the margin of the tumor and the peripheral healthy tissue.

There are two major consequences of this evidence. First, different pathways favoring tumor growth, invasion and immune escape are present in different regions of the tumor. Second, the removal of the ALA-PALE region is fundamental for the eradication of a microenvironment capable of promoting the growth of any residual tumor cells.

However, these data, although fascinating, derive from in vitro study. For this reason, we decided to assess their possible clinical relevance. Since there are no specific transcriptomic studies on the infiltrating front of GBM, we compared our results with data collected from 163 GBM included in the Cancer Genome Atlas GBM dataset (TCGA-GBM) and 207 TCGA/GTEx matched normal samples. Interestingly, eleven of the ALA+/ALA− differentially expressed genes (ACKR3, CCL2, CXCL11, CXCL2, CXCL8, CXCR4, EGFR, IL1B, SPP1, TGFB1 and CCR1) were also upregulated in tumor patients, with respect to matched normal samples, and four others (CXCL1, CXCL5, CD274 and PTGS2) showed a similar, although not statistically significant, trend. Five of these genes (CCL2, CXCL2, CXCL8, SPP1 and TGFB1) resulted to be prognostic for both OS and DFS. Importantly, three of these genes (CXCL2, CXCL8 and SPP1) were indeed upregulated also in the microenvironmental cells deriving from the ALA-PALE area.

The fact that GASC retain a transcriptional profile resembling the tissue of origin remains an interesting issue. Previous genetic studies excluded that this phenomenon could be attributed to genetic mutations, since GASC are devoid of genetic mutations, and suggested that some epigenetic changes could be responsible for the maintenance of GASC features in culture [30]. Regarding the mechanisms responsible for these changes we have previously shown that GASC are able to increase proliferation, motility and anchorage independent growth of both commercially available glioblastoma cell lines and GSC isolated from the same tumors through the release of exosomes [30]. We identified a protein, carried on the surface of GASC-exosomes (SEMAPHORIN-7A), that signals to GSC through integrin-β1, activating focal adhesion kinase and increasing GSC motility, in vitro [86]. On the other side, we also observed that exosomes released in the glioma microenvironment by GSC regulate the immunomodulatory properties of peripheral immune cell populations and promote the tumor immune escape [87]. These findings support the existence of a bi-directional crosstalk between glioma cells and their surrounding microenvironment: glioma cells co-opt the tumor microenvironment inducing changes in their phenotypes and their ability to support tumor growth while the microenvironment releases pro-tumorigenic signals. In the present study, we studied the properties of microenvironments mapping at different distances from the GBM core (GASC-ALA+, GASC ALA-PALE and GASC-ALA−) and we found different pathways related to tumor growth, infiltration and immune tolerance in the different regions of the tumor. Further studies are required to elucidate the mechanisms responsible for these differences.

In conclusion, 5-ALA-guided surgery in combination with the study of GASC made it possible to highlight that the crosstalk between tumor cells and the microenvironment is not uniform throughout the tumor. In fact, tumor growth, infiltration and immune tolerance are supported by different pathways in different regions of the tumor.

## 4. Materials and Methods

### 4.1. Intraoperative Tumor Sampling

Tumor tissues were collected from patients affected by a supratentorial GBM arising *de novo*. All patients, not previously treated with neo-adjuvant therapy, underwent surgical resection of the tumor at the Neurosurgical Department of the Azienda Ospedaliera Universitaria of Udine or at the Fondazione Policlinico Universitario Agostino Gemelli IRCCS (Rome, Italy). The independent ethic committee of both the Azienda Ospedaliero-Universitaria of Udine (Parere 196/2014/Em) and of the Fondazione Policlinico Universitario Agostino Gemelli (Prot. N. 0020786/18) has approved the research. Written informed consents have been obtained from patients and all clinical investigations have been conducted according to the principles expressed in the Declaration of Helsinki.

5-ALA (20 mg/kg body weight; Medac GmbH, Wedel, Germany) was administered orally to patients 3–5 h prior to neurosurgery. At the time of surgery, three different tumor macro-areas were visualized based on the presence of red-violet fluorescence, following excitation with ultraviolet blue light (FL 400 filter, Leica M720 OH5; Leica Microsystems, Wetzlar, Germany).

Sampling sites were checked with intraoperative neuro-navigation (based on preoperative contrast enhanced MRI) and categorized as follows:

(1) Tumor core with lava-like red fluorescence (ALA+ area), corresponding to the enhancing area at navigation.
(2) Tumor inner periphery with pale red fluorescence (ALA PALE area), corresponding to the tumoral boundaries directly surrounding the enhancing area.
(3) Tumor outer periphery without fluorescence (ALA− area), corresponding to non-enhancing areas at navigation at a distance from the tumor.

A few mm^3^ of tissue were collected on solid non-necrotic areas and preserved in Dulbecco’s Modified Eagle Medium (DMEM; Invitrogen, Carlsbad, CA, USA,) at 4 °C, before processing.

### 4.2. Isolation and Culture of Glioma Associated Stem Cells (GASC)

Glioma-associated stem cells (GASC) were isolated from the three regions, displaying a progressive intensity of fluorescence for 5-aminolevulinc acid and maintained, in vitro, applying, with minor modifications, a protocol optimized for culturing multipotent adult stem cells from normal and neoplastic human tissues. Briefly, GBM fragments were first mechanically disaggregated with scalpels and then enzymatically dissociated, in a 0.025% Collagenase type II solution (Worthington) in Joklik modified Eagle’s Medium (Sigma-Aldrich, St.Louis, MO, USA) for 5 min at 37 °C. Collagenase activity was stopped by adding 10% Fetal Bovine Serum in Joklik modified Eagle’s Medium (Sigma-Aldrich). Cell suspension was centrifuged at 500× *g* for 10 min after being filtered through a sieve (BD Falcon, Franklin Lakes, NJ, USA) in order to select a population less than 40 μm in diameter. 2.0 × 10^6^ freshly isolated human cells were plated onto 100 mm diameter, human fibronectin (Sigma-Aldrich)-coated dishes (BD Falcon), in an expansion medium composed as follows: 60% low glucose DMEM (Invitrogen), 40% MCDB-201, 1 mg/mL linoleic acid-BSA, 10^−9^ M dexamethasone, 10^−4^ M ascorbic acid-2 phosphate, 1× insulin-transferrin-sodium selenite (all from Sigma-Aldrich), 2% foetal bovine serum (StemCell Technologies, Cambridge, UK), 10 ng/mL human PDGF-BB, 10 ng/mL human EGF (both from Peprotech EC, London, UK).

### 4.3. Histology

Alongside with cell isolation, all the resected tissue samples (ALA+, ALA PALE and ALA−) were formalin fixed, paraffin embedded, and processed for routine histopathological and immunohistochemical staining. Histological examination, immunohistochemistry (IHC) for GFAP, Ki67, p53 and ATRX and assessment of IDH1/2 genes mutation were performed as previously reported [30,33,88].

### 4.4. Flow-Cytometry

Once reached the third passage in culture, GASC were detached using TRYPLE Express (Invitrogen) and incubated with properly conjugated primary antibodies recognizing: CD29, CD49a, CD49b, CD49d, CD9, CD144, CD44, CD117, CD59, CD38 (BD Biosciences, San Jose, CA, USA) CD73, CD90, CD34 (eBioscience, Paris, France), CD271, CD34, CD325 (BD Pharmigen, San Jose, CA, USA), CD105, CD66e (Serotech, Milano, Italy), CD133, CD271 (Miltenyi Biotec, Bergisch Gladbach, Germany), CD105 (Santa Cruz Biotechnology, Dallas, TX, USA), ABCG-2, CXCR4 (R&D), CD49f (BioLegend, San Diego, CA, USA), CD10 (AbD Serotec, Milano, Italy). Properly conjugated isotype matched antibodies were used as negative control. At least 2 × 10^4^ events were collected by FACS CANTO II (BD-Biosciences, San Jose, CA, USA) and analyzed using FlowJo (Becton Dickinson, San Jose, CA, USA) software.

### 4.5. Immunofluorescence

Cells were cultured in expansion medium until third passage and, once seeded in 24-well plates at a density of 10,000 cell/cm^2^, they were fixed in 4% paraformaldehyde in PBS for 20 min at room temperature (RT). For intracellular staining, fixed cells were permeabilized for 10 min at RT with 0.1% (to detect intermediate filaments protein) or 0.3% (to detect nuclear markers) Triton X-100 (Sigma-Aldrich, St Louis, MO, USA) in PBS. Unspecific binding was blocked using 5% BSA, in PBS, for 1h at RT, and cells were incubated overnight at 4 °C to detect the following proteins: Oct-4 (1:100, Abcam, Cambridge, UK); Sox-2 (1:200, Abcam); nestin (1:100, Millipore, Burlington, MA, USA); GFAP (1:50, Dako, Glostrup, Denmark); vimentin (1:500, Dako); neural/glial antigen 2 (NG2, 1:100, Invitrogen, Carlsbad, CA, USA); T box transcription factor 15/18 (TBX 15/18, 1:100, Invitrogen). A488- and A555- conjugated secondary antibodies, diluted 1:800, for 1 h at 37 °C, were employed. Vectashield (Vector Labs, Burlingame, CA, USA) added with 0.1 μg/mL DAPI (Sigma, St Louis, MO, USA) was used as mounting medium. Images were processed by a confocal microscope (Leica TCS-SP2 or Leica TCS-SP8, Leica Microsystems, Wetzlar, Germany, http://leica.com).

### 4.6. RNA Extraction and cDNA Synthesis

Total RNA was extracted using the RNAeasy mini kit (Qiagen, Hilden, Germany), according to manufacturer’s instructions. Next, cDNA was synthesized using the RT2 First Strand Kit (Qiagen, Hilden, Germany) following the manufacturer’s instructions.

### 4.7. RT2 Profiler™ PCR Array

Eighty-four genes or biological pathways involved in mediating inflammation and immunity were analyzed using the RT2 Profiler Cancer Inflammation and Immunity Crosstalk PCR Array (PAHS-181Z; Qiagen S.r.l.). According to the manufacturer’s protocol, real-time PCR was performed using RT2 Profiler PCR Arrays in combination with RT2 SYBR Green/ROX PCR Master Mix (Qiagen). A 102-μL cDNA synthesis reaction volume was mixed with 2 × RT2 SYBR Green Mastermix and RNase-free water to obtain a total volume of 2700 μL. Subsequently, 25 μL of the PCR component mix was dispensed into each of the 96-well PCR array. The cycling program, performed on Roche LightCycler 480, comprises three steps: 95 °C for 10 min for 1 cycle, and 45 cycles at 95 °C for 15 s and 60 °C for 60 s. Data analyses were performed using the manufacturer’s software (https://geneglobe.qiagen.com/us/analyze/), which calculates the fold change/regulation of the investigated genes using delta Ct (∆Ct) method. Briefly, ∆Ct is calculated between each gene and an average of the housekeeping genes (*ACTB*, *B2M*, *GAPDH*, *HPRT1*, and *RPLP0*). Then, ∆∆Ct was extrapolated as the difference between ∆Ct of genes in the tested group (ALA+, ALA PALE) and ∆Ct of the same genes in the control group (ALA−). Finally, fold change is calculated using 2^ (−∆∆Ct) formula.

### 4.8. Differential Gene Expression Analysis in the TCGA-GBM Dataset

The differentially expressed genes identified with the RT2 Profiler array in the ALA+/ALA PALE/ALA− comparisons were also tested comparing their expression levels in the TCGA-GBM dataset (*n* = 163) with respect to the TCGA-GTEx matched normal samples (*n* = 207). Data were obtained from the GEPIA web server (http://gepia.cancer-pku.cn/index.html, last accessed March 2020) and summarized as boxplots; |Log2FC| Cutoff: 1; *p*-value Cutoff: 0.01.

### 4.9. Survival Analysis

Genes differentially expressed according to both the RT2 profiler array and the GEPIA web server underwent a two-steps survival analysis. First, we defined the prognostic value in the TCGA-GBM dataset on a single gene basis in terms of overall survival (OS) and disease-free survival (DFS). Afterwards, we defined a five-gene signature and tested it in the same way. We censored data at 24 months for OS and at 12 months for DFS.

Gene expression data (RSEM-normalized RNA-seq data) were downloaded from http://firebrowse.org/?cohort=GBM&download_dialog=true. Clinical data were downloaded from http://www.cbioportal.org/study?id=gbm_tcga#clinical. The R/Bioconductor survminer package was used to determine the optimal cut-point for continuous variables and the division of patients was done within the 25–75% percentile range of gene expression. The R/Bioconductor RTCGA clinical and survival packages were used to calculate hazard ratios and confidence intervals and to draw the associated Kaplan-Meier plots.

## 5. Conclusions

5-ALA FGS allowed insights into the heterogeneity of the glioma microenvironment demonstrating that ALA+, ALA-PALE and ALA− regions were characterized by a tumor-supporting signature, although subtended by different pathways. Deepening knowledge of these differences can be instrumental to develop new adjuvant therapies properly targeting the crosstalk between tumor cells and their supporting microenvironment.

## Figures and Tables

**Table 1:** Comparative expression of surface antigens evaluated by flow cytometry. The fraction of cells expressing the assayed markers is reported as mean ± SD. GASC-ALA+ (*n* = 3), GASC ALA PALE (*n* = 3) and GASC-ALA− (*n* = 3) were analyzed at the third passage in culture.

MARKER	GASC ALA+	GASC ALA PALE	GASC ALA−
**CD59**	94.80 ± 2.34	92.58 ± 4.62	92.45 ± 2.99
**CD29**	92.81 ± 6.77	98.93 ± 0.47	97.13 ± 1.96
**CD49B**	90.5 ± 7.08	96.53 ± 1.28	90.90 ± 6.60
**CD49D**	87.95 ± 3.40	87.51 ± 1.28	82.91 ± 5.67
**CD9**	84.43 ± 5.16	87.46 ± 2.36	73.89 ± 9.63
**CD44**	82.74 ± 10.53	92.73 ± 1.29	88.07 ± 5.23
**CD73**	81.05 ± 12.21	90.41 ± 5.20	94.32 ± 2.65
**CD105**	82.9 ± 1.55	61.86 ± 11.61	86.26 ± 6.71
**CD10**	81.82 ± 10.25	97.24 ± 1.86	86.87 ± 5.01
**CD49F**	79.64 ± 5.67	75.7 ± 0.57	63.66 ± 8.39
**CD49A**	74.47 ± 19.25	83.06 ± 7.72	85.06 ± 2.38
**CD90**	66.65 ± 19.28	31.12 ± 17.7	19.60 ± 9.78
**ABCG2C**	2.47 ± 2.25	0.27 ± 0.12	0.10 ± 0.22
**CXCR4**	1.20 ± 0.85	0.47 ± 0.15	0.1 ± 0.1
**CD144**	3.23 ± 5.43	0.17 ± 0.4	1.28 ± 0.26
**CD133**	0.4 ± 0.52	0.17 ± 0.24	0.05 ± 0.05
**CD117**	0	0.13 ± 0.15	0.16 ± 0.27
**CD34**	0	0.01 ± 0.02	0.43 ± 0.25
**CD271**	0	0	0
**CD38**	0	0	0

**Table 2:** Comparison of genes differentially expressed in GASC-ALA+ versus GASC-ALA−. Table show genes with a significant up or down-regulation, in GASC-ALA+ compared with GASC-ALA−. Values indicate Fold Regulation calculated using the formula (2^ (−Delta Delta CT)), i.e., the normalized gene expression (2^ (−Delta CT)) in the test sample (GASC-ALA+) divided by the normalized gene expression (2^ (−Delta CT)) in the control ample (GASC-ALA−). *p* values are calculated based on a Student’s t-test of the replicate 2^ (−Delta CT) values for each gene in the control and treatment group.

GENE SYMBOL	GENE NAME	PROTEIN FUNCTION	FOLD ALA+ vs. ALA−	*p*-Value
**SPP1**	Secreted phosphoprotein-1/Osteopontin	Modulator of interferon gamma gene expression	19.58	0.004
**CXCR4**	Chemokine (C-X-C motif) receptor 4	Chemokine receptor	16.3	0.002
**CSF3**	Colony stimulating factor 3	Growth factor for Granulocytes	8.13	0.021
**CCL2**	Chemokine (C-C motif) ligand 2	Pro-inflammatory factor	6.3	0.011
**CXCL8**	Interleukin 8	Immunosuppressive factor	6.29	0.0001
**IL1B**	Interleukin 1. beta	Pro-inflammatory cytokine	5.2	0.02
**CXCL5**	Chemokine (C-X-C motif) ligand 5	Neutrophil activating peptide	4.55	0.007
**PTGS2**	Prostaglandin-endoperoxide synthase	Immunosuppressive factor	4.49	0.015
**CXCL11**	Chemokine (C-X-C motif) ligand 11	Interferon inducible T-cell chemoattractant	3.6	0.014
**ACKR3**	Atypical chemokine receptor-3	Chemokine receptor	3.55	0.01
**CXCL1**	Chemokine (C-X-C motif) ligand 1	Chemotactic for Neutrophils	3.03	0.03
**VEGFA**	Vascular endothelial growth factor A	Pro-inflammatory factor	2.86	0.054
**CD274**	Programmed death ligand 1 (PDL-1)	Immunosuppressive factor	2.81	0.019
**CXCL2**	Chemokine (C-X-C motif) ligand 2	Macrophage inflammatory protein-2 alpha	2.69	0.03
**TGFB1**	Transforming growth factor. beta 1	Immunosuppressive factor	2.05	0.042
**EGFR**	Epidermal growth factor receptor	Cell proliferation inducing protein	2.04	0.009
**CCL28**	Chemokine (C-C motif) ligand 28	CD4/CD8 T-Cell chemotactic protein	−3.59	0.017
**CCR7**	Chemokine (C-C motif) receptor 7	Chemokine receptor	−3.21	0.174
**CCR10**	Chemokine (C-C motif) receptor 10	Chemokine receptor	−2.64	0.011

**Table 3:** Comparison of genes differentially expressed in GASC-ALA+ versus GASC-ALA PALE. Table shows genes, with a significant up or down-regulation, in GASC-ALA+ compared to GASC-ALA PALE. Values indicate Fold Regulation calculated using the formula (2^ (−Delta-Delta CT)), i.e., the normalized gene expression (2^ (−Delta CT)) in the test sample (GASC-ALA+) divided by the normalized gene expression (2^ (−Delta CT)) in the control sample (GASC-ALA PALE). *p*-values are calculated based on a Student’s t-test of the replicate 2^ (−Delta CT) values for each gene in control and treatment groups.

GENE SYMBOL	GENE NAME	PROTEIN FUNCTION	FOLD ALA+ vs. ALA PALE	*p*-Value
**CXCR4**	Chemokine (C-X-C motif) receptor 4	Chemokine receptor	25	0.002
**ACKR3**	Atypical chemokine receptor 3	Chemokine receptor	6.85	0.003
**CXCL11**	Chemokine (C-X-C motif) ligand 1	Interferon inducible T-cell chemoattractant	4.19	0.058
**CCL2**	Chemokine (C-C motif) ligand 2	Pro-inflammatory factor	3.52	0.015
**SPP1**	Secreted phospoprotein1/Osteopontin	Modulation of interferon gamma gene expression	3.47	0.037
**CD274**	Programmed death ligand 1 (PDL-1)	Immunosuppressive factor	2.46	0.02
**BCL2**	B-Cell CLL/Lymphoma 2	Antiapoptotic factor	2.28	0.003
**CXCL12**	Chemokine (C-X-C motif) ligand 1	Immunosuppressive factor	2.15	0.738
**PTGS2**	Prostaglandin-endoperoxide synthase	Immunosuppressive factor	2.12	0.8
**CCL20**	Chemokine (C-C motif) ligand 20	Chemotactic for Dendritic/T and B-Cells	−12.39	0.13
**CCL28**	Chemokine (C-C motif) ligand 28	CD4/CD8 T-Cells chemotactic protein	−4.75	0.034
**IL1B**	Interleukin 1. beta	Pro-inflammatory cytokine	−3.41	0.149
**CXCL1**	Chemokine (C-X-C motif) ligand 1	Chemotactic for Neutrophils	−3.1	0.136
**CSF3**	Colony stimulating factor 3	Growth factor for Granulocytes	−2.45	0.177
**CCR7**	Chemokine (C-C motif) receptor 7	Chemokine receptor	−2.42	0.84
**CCR1**	Chemokine (C-C motif) receptor 1	Chemokine receptor	−2.28	0.025
**CCR10**	Chemokine (C-C motif) receptor 10	Chemokine receptor	−2.09	0.012

**Table 4:** Comparison of genes differentially expressed in GASC-ALA PALE versus GASC-ALA−. Table shows genes with a significant up- or down-regulation, in GASC-ALA PALE compared to GASC-ALA−. Values indicate Fold Regulation calculated using the formula (2^ (−Delta Delta CT)), i.e., the normalized gene expression (2^ (−Delta CT)) in the test sample (GASC-ALA PALE) divided by the normalized gene expression (2^ (−Delta CT)) in the control sample (GASC-ALA−). *p* values are calculated based on a Student’s t-test of the replicate 2^ (−Delta CT) values for each gene in control and treatment groups.

GENE SYMBOL	GENE NAME	PROTEIN FUNCTION	FOLD ALA PALE vs. ALA−	*p*-Value
**CSF3**	Colony stimulating factor 3	Growth factor for Granulocytes	19.9	0.168
**IL1B**	Interleukin 1, beta	Pro-inflammatory cytokine	17.74	0.108
**CCL20**	Chemokine (C-C motif) 20	Chemotactic for Dendritic/T and B-Cells	13.06	0.134
**CXCL1**	Chemokine (C-X-C motif) ligand 1	Chemotactic for Neutrophils	9.4	0.091
**CXCL8**	Interleukin 8	Immunosuppressive factor	7.26	0.112
**SPP1**	Secreted phosphoprotein 1/Osteopontin	Modulator of interferon gamma gene expression	5.63	0.09
**CXCL5**	Chemokine (C-X-C motif) ligand 5	Neutrophil activating peptide	5.49	0.148
**CXCL2**	Chemokine (C-X-C motif) ligand 2	Macrophage inflammatory protein-2 alpha	5.28	0.105
**PTGS2**	Prostaglandin-endoperoxide synthase	Immunosuppressive factor	2.12	0.163
**IL1A**	Interleukin 1, alpha	Proinflammatory cytokine	2.09	0.052
**CSF2**	Colony stimulating factor 2	Stimulating for Ganulocyte and Macrophages	2.06	0.06
**CXCL12**	Chemokine (C-X-C motif) ligand 1	Immunosuppressive factor	−2.26	0.144

**Table 5:** Univariate analysis in terms of disease-free survival (DFS) and overall survival (OS). The Cox regression analysis was performed for the five genes considered either singularly or included in a five-gene signature. Hazard ratios and confidence intervals were calculated using the survival R/Bioconductor package.

Variable	Patients Number	HR	95% C.I.	*p*-Value
**DFS (12 months)**				
CCL2 expression	High (*n* = 44) Low (*n* = 74)	1 0.54	0.33–0.88	0.012
CXCL2 expression	High (*n* = 40) Low (*n* = 78)	1 0.42	0.26–0.68	<0.01
CXCL8 expression	High (*n* = 42) Low (*n* = 76)	1 0.58	0.36–0.94	0.029
SPP1 expression	High (*n* = 81) Low (*n* = 37)	1 0.56	0.32–0.96	0.034
TGFB1 expression	High (*n* = 37) Low (*n* = 81)	1 0.61	0.37–1	0.049
Five gene signature expression	High (*n* = 78) Low (*n* = 40)	1 0.56	0.33–0.95	0.03
**OS (24 months)**				
CCL2 expression	High (*n* = 103) Low (*n* = 61)	1 0.63	0.43–0.94	0.022
CXCL2 expression	High (*n* = 60) Low (*n* = 104)	1 0.56	0.38–0.81	0.002
CXCL8 expression	High (*n* = 90) Low (*n* = 74)	1 0.76	0.52–1.1	0.153
SPP1 expression	High (*n* = 128) Low (*n* = 36)	1 0.62	0.39–1	0.052
TGFB1 expression	High (*n* = 51) Low (*n* = 113)	1 0.63	0.43–0.93	0.021
Five gene signature expression	High (*n* = 111) Low (*n* = 53)	1 0.64	0.42–0.96	0.03
